# Characterization of a Highly Virulent *Edwardsiella anguillarum* Strain Isolated From Greek Aquaculture, and a Spontaneously Induced Prophage Therein

**DOI:** 10.3389/fmicb.2019.00141

**Published:** 2019-02-06

**Authors:** Pantelis Katharios, Panos G. Kalatzis, Constantina Kokkari, Michail Pavlidis, Qiyao Wang

**Affiliations:** ^1^Institute of Marine Biology, Biotechnology and Aquaculture, Hellenic Centre for Marine Research, Heraklion, Greece; ^2^Marine Biological Section, University of Copenhagen, Copenhagen, Denmark; ^3^Department of Biology, University of Crete, Heraklion, Greece; ^4^State Key Laboratory of Bioreactor Engineering, East China University of Science and Technology, Shanghai, China; ^5^Shanghai Engineering Research Center of Maricultured Animal Vaccines, Shanghai, China

**Keywords:** *Edwardsiella anguillarum*, *Diplodus puntazzo*, comparative genomics, virulence, sequencing, prophage, lysogenic conversion, motility

## Abstract

*Edwardsiella*-associated outbreaks are increasingly reported on both marine and freshwater aquaculture setups, accounting for severe financial and biomass losses. *E. tarda, E. ictaluri*, and *E. hoshinae* have been the traditional causative agents of edwardsiellosis in aquaculture, however, intensive studies due to the significance of the disease have just recently revealed two more species, *E. piscicida* and *E. anguillarum*. Whole genome sequencing that was conducted on the strain EA011113, isolated from farmed *Diplodus puntazzo* after an edwardsiellosis outbreak in Greece, confirmed it as a new clinical strain of *E. anguillarum*. Extensive phylogenetic analysis showed that this Greek strain is closely related to an Israeli *E. piscicida-*like clinical strain, isolated from diseased groupers, *Epinephelus aeneus* and *E. marginatus* in Red Sea. Bioinformatic analyses of *E. anguillarum* strain EA011113 unveiled a wide repertoire of potential virulence factors, the effect of which was corroborated by the mortalities that the strain induced in adult zebrafish, *Danio rerio*, under different levels of infection intensity (LD_50_ after 48 h: 1.85 × 10^4^ cfu/fish). This strain was non-motile and according to electron microscopy lacked flagella, a fact that is not typical for *E. anguillarum*. Comparative genomic analysis revealed a deletion of 36 nt found in the flagellar biosynthetic gene (*FlhB*) that could explain that trait. Further *in silico* analysis revealed an intact prophage that was integrated in the bacterial genome. Following spontaneous induction, the phage was isolated, purified, characterized and independently sequenced, confirming its viability as a free, inducible virion as well. Separate genomic analysis of the prophage implies a plausible case of lysogenic conversion. Focusing on edwardsiellosis as a rapidly emerging aquaculture disease on a global scale, this work offers some insight into the virulence, fitness, and potential lysogenic conversion of a of a newly described, yet highly pathogenic, strain of *E. anguillarum*.

## Introduction

Edwardsiellosis is a serious disease affecting a wide range of cultured fish species both in marine and freshwater environments. It is increasingly becoming a threat for the viability of aquaculture industries worldwide (Xu and Zhang, 2014) and its main causative agents are *Edwardsiella tarda*, *E. ictaluri*, *E. piscicida*, and *E. anguillarum* (Shao et al., 2015). Recently, we described the first incidence of edwardsiellosis in the Mediterranean Sea, in cultured sharpsnout seabream, *Diplodus puntazzo* (Katharios et al., 2015). At this incidence we had identified the isolates as *E. tarda*, based on their phenotypic characteristics and 16s rRNA sequences. However, due to the highly conserved rRNA genes, differences cannot be detected between closely related species. The use of high resolution phylogenetic methods such as multilocus sequence analysis (MLSA), multilocus sequence typing (MLST) and/or whole genome sequencing (Shao et al., 2015) is required in order to properly classify novel members of *Edwardsiella* spp. Here, we analyzed the strain EA011113 which was isolated in Greece, using whole genome sequencing, focusing on its phylogenetic position, presence of prophages, virulence factors and motility. We studied the virulence of the isolate *in vivo*, using zebrafish, and *in silico*, by analysis of virulence-related genes. We showed that the strain belonged to the newly described species *E. anguillarum* (Shao et al., 2015) and was closely related to the *E. piscicida*-like strain isolated from diseased groupers in the Red Sea (Ucko et al., 2016). Furthermore, the isolate contained a spontaneously inducible intact prophage that is genetically similar to both a prophage found in the bacterial genome of an *E. piscicida*-like strain EA181011 (Reichley et al., 2015) and the free bacteriophage GF-2 (Yasuike et al., 2015) that has been isolated against *E. tarda*.

## Materials and Methods

### Bacterial Strains Used

The *E. anguillarum* strain EA011113 was isolated from diseased sharpsnout seabream, *Diplodus puntazzo*, in Greece (Katharios et al., 2015), while *E. anguillarum* type strain ET080813 was isolated from diseased eel, *Anguilla anguilla*, in China (Wang Q. et al., 2009). Both strains were stored in microbeads (BIOBANK) in −80°C and were grown overnight at 28°C in Luria-Bertani (LB) medium or Brain Heart Infusion Agar (BHIA) plates.

### Phenotypes Description and Prophage Induction

Motility of the bacterial strains was assessed following inoculation in Motility Test Medium (Motility Indole Orninthine Medium-Sigma Aldrich). Motility was visualized as a diffused zone of growth flaring out from the line of inoculation.

The EA011113 prophage was spontaneously released after overnight culture of the bacteria in BHI broth, at 25°C. Serial dilutions of the overnight culture filtrate (through 0.2 μm syringe filter) was mixed with fresh culture at early exponential phase (∼10^7^ cfu/ml) and subsequently with soft agar and plated on BHI bottom agar plates (double layer assay) resulting in the formation of lytic plaques. One of these plaques was selected and left at 4°C in SM buffer (50 mM Tris-HCl, pH7.5, 100 mM NaCl, 8 mM MgSO4, 0.01% gelatin) for 20 h. Then the phage particles were separated from agar and bacteria by filtration and this procedure was repeated 5 times in order to purify the phage. The titer of the phage was measured by the double layer assay.

For TEM observation and DNA extraction an increase of the initial titer by one order of magnitude was achieved by precipitation with Polyethylene glycol (20% PEG-8000/2.5 M NaCl) (Yu and Ferretti, 1991).

### Sequencing and Genomic Analysis

Paired-end sequencing was performed for both bacterium and the isolated phage, using an Illumina MiSeq platform (Illumina, San Diego, CA, United States). Sequencing process, library construction and trimming of contaminated reads and primers, were performed in accordance with the manufacturer’s protocols. *De novo* assembly of the obtained reads was done by Masurca assembler (Zimin et al., 2013) producing single contigs, whereas short and low-coverage contigs were discarded. Quality of the assembled genome sequences was assessed by Benchmarking Universal Single-Copy Orthologs (BUSCO) (Sima et al., 2015). Further processing of the genomes as well as their final submission to the National Center for Biotechnology Information (NCBI) was conducted through Geneious software bioinformatics platform R10 version (Kearse et al., 2012).

Bacterial genes identification and annotation were done using the NCBI Prokaryotic Genome Annotation Pipeline (Tatusova et al., 2016) and Rapid Annotation Subsystem Technology (RAST) (Aziz et al., 2008). Complementary analysis of the bacterial virulence genes was performed by the Pathosystems Resource Integration Center platform (PATRIC), (Wattam et al., 2013) which combines 3 databases for the identification of virulence related-genes; PATRIC-VF, VFDB, and VICTORS. Special focus was on T3SS and T6SS which were studied using alignment with the respective gene clusters identified in the strain ET080813 and strain EA011113. Differences in motility were studied by comparing sequences of the flagellar clusters found in the non-motile strains EA011113 and the Israeli *Edwardsiella piscicida*-like strain EA181011, to the respective clusters of the motile Chinese *E. anguillarum* type strain ET080813.

The genes of the sequenced bacteriophage were *in silico* identified using Glimmer 3 (Delcher et al., 2007) and the potential coding sequences (CDSs) were generated. Rapid Annotation Subsystem Technology (RAST) (Aziz et al., 2008; Overbeek et al., 2013) was used in order to annotate the predicted genes. CDSs of both annotated and hypothetical function were crosschecked manually by protein Basic Local Alignment Tool (BLASTP) (McGinnis and Madden, 2004) and by Protein Fold Recognition Server, Phyre2 (Kelley et al., 2015), defining the gene functions both by genetic similarity and by protein structure. The presence of tRNAs was assessed by the online bioinformatic tool ARAGORN (Laslett and Canback, 2004). The determination of phage termini and packaging mechanism of the virus was attempted by using PhageTerm (Garneau et al., 2017), inserting the raw reads phage sequencing data. VIRFAM server (Lopes et al., 2014) based on the recognition of head-neck-tail modules in phage genomes, was used in order to classify Edno5 genome according to remote homology detection of its viral protein families.

The tertiary protein structures presented in the results were configured by InterProScan (Jones et al., 2014) sequence analysis application, through combination of different protein signature recognition methods into one resource.

### Accession Numbers

The contigs of *E. anguillarum* EA011113 genome as well as the genome of its temperate phage Edno5 were deposited to NCBI under the accession numbers PRJNA393918 and MH898687, respectively. The sequence of the phage is also contained in contig 113 with accession code NMPN01000113.1 that belongs to the previously mentioned BioProject PRJNA393918.

### Phylogenetic Analysis

The nucleotide level genomic similarity (%) of EA011113 genome with other *Edwardsiella* spp. genomes was studied using Average Nucleotide Identity by Orthology (Lee et al., 2016). The genome of the *Edwardsiella* isolate of the current study (EA011113) was compared to the genomes of EA181011 (GenBank: CP011364.1), an *E. piscicida*-like strain isolated from diseased grouper in Eilat, Israel (Ucko et al., 2016), ET080813 (Genbank: CP006664.1), isolated from diseased eels in China which is the type strain of the newly proposed species, *Edwardsiella anguillarum* (Shao et al., 2015), *E. piscicida* strain C07_087 (Genbank: NC_020796.1), *E. ictaluri* strain MS-17-165 (Genbank: CP028813.1), *E. tarda* strain FL95-01 (Genbank: CP011359.1) and *E. hoshinae* strain ATCC35051 (Genbank: CP016043.1). The Average Nucleotide Identity tool developed by Konstantinidis lab (Rodriguez-R and Konstantinidis, 2016) was also used to compare the genome of EA011113 to EA181011 and ET080813.

### Multilocus Sequencing Typing (MLST)

Multilocus sequencing typing allele sequences and ST (sequence type) profile tables for *Edwardsiella* spp. hosted at the University of Oxford database^1^ were used for the analysis (Buján et al., 2018a). The specific method uses 10 genetic loci (*Adk*, *AtpD*, *DnaJ*, *GapA*, *GlnA*, *Hsp60*, *PhoR*, *PyrG*, *RpoA*, and *Tuf*) and has 18 different STs. The allele sequences from all 62 strains of the databases (April 2018) were retrieved and aligned with the respective sequences of EA011113 and EA181011 in Geneious R9.1.8. Following trimming, the alignment of the concatenation of these sequences was used to construct a phylogenetic tree using the Neighbor-Joining method with 1000 bootstraps in MEGA7.

### *In vivo* Virulence

Adult zebrafish (mean weight: 0.3 g) were used for assessing the virulence of EA011113. 70 fish were distributed in seven 5-L tanks each containing 10 individuals. Six doses of live bacteria following 10-fold serial dilutions in sterile saline were injected in the experimental groups (10^7^–10^2^ cfu per fish) while the control group was injected with sterile saline. The injection was done using a Hamilton microsyringe following anesthesia (MS222), while the injection volume was 10 μL per fish. The fish were monitored over a 5-day period, dead fish were removed daily and mortalities were recorded. LD_50_ value was estimated by the dose-response curve at 48 h post injection using probit analysis (Finney and Tattersfield, 1952). The procedure was performed at the University of Crete which has licensed designated facilities for experimentation with animals (registration number: EL-BIOexp-10) and the protocol was approved by the General Directorate of Regional Agricultural Economy and Veterinary Services of the Region of Crete (License number: 147115/17-07-2017).

### Transmission (TEM) and Scanning (SEM) Electron Microscopy

Bacterial (EA011113 and ET080813) and phage (Edno5) samples were preserved in 2.5% Glutaraldehyde in phosphate buffer for scanning electron microscopy (SEM) and transmission electron microscopy (TEM). Samples for SEM were washed with sodium cacodylate buffer, post fixed with OsO_4_ and dehydrated in an ascending alcohol series, mounted on stubs, and sputter coated with gold-palladium. Bacteria were viewed using a JEOL JSM-6390LV scanning electronic microscope at 20 kV. Samples for TEM were negatively stained with 4% (w/v) uranyl acetate (pH 7.2) and observed with a JOEL JEM2100 operated at 80 kV at the Electron Microscopy Laboratory of the University of Crete. Bacteria were grown for 6 h in TSB before observations.

## Results and Discussion

### Genomic Description of *E. anguillarum* Strain EA011113

Following *de novo* assembly, sequencing of EA011113 resulted in 139 contigs (N50: 174066, maximum contig length 430,834 bp). The total sequence length was 4,031,776 bp with a GC content of 59.12%. According to NCBI Prokaryotic Genome Annotation Pipeline the genome contained 3610 CDS, 143 RNA genes, 43 rRNAs, 94 tRNAs, and 153 pseudogenes. This pipeline was used for the final submission of EA011113 genome in NCBI. PATRIC analysis and annotation resulted in a genome of 3,959,109 bp containing 3805 CDS, 15 rRNAs and 86 tRNAs. RAST analysis assigned 293 genes to cofactors, prosthetic groups and pigments, 190 genes to cell wall and capsule, 74 to virulence, 30 to potassium metabolism, 9 to phages, prophages, transposable elements, plasmids, 134 to membrane transport, 22 to iron acquisition, 203 to RNA metabolism, 248 to protein metabolism, 37 to cell division and cell cycle, 84 to motility and chemotaxis, 108 to regulation and cell signaling, 4 to secondary metabolism, 122 to DNA metabolism, 111 to fatty acids, lipids and isoprenoids, 39 to nitrogen metabolism, 3 to dormancy and sporulation, 146 to respiration, 133 to stress response, 22 to metabolism of aromatic compounds, 377 to amino acids and derivatives, 19 to sulfur metabolism, 38 to phosphorus metabolism and 405 to carbohydrates.

### Phylogenetic Analysis Reclassifies EA011113 as *E. anguillarum*

**FIGURE 1:**
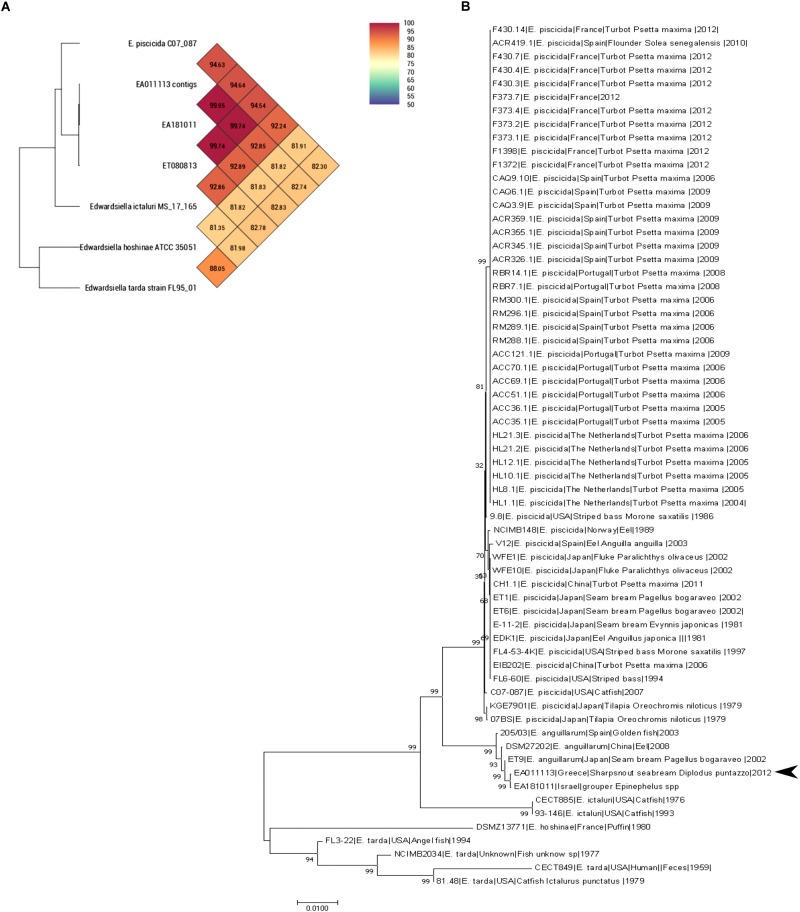
**(A)** OrthoANI values (% nucleotide level genomic similarity) of the EA011113 against other *Edwardsiella* spp. **(B)** MLST analysis of all available *Edwardsiella* species in the BIGSdb. Phylogenetic tree was build using Neighbor-joining method with 1000 bootstraps. EA011113 forms a distinct cluster with the other *E. anguillarum* strains. There were a total of 6617 positions in the final dataset.

Both Average Nucleotide Identity by Orthology (Lee et al., 2016) and Average Nucleotide Identity tool developed by Konstantinidis lab showed that EA011113 is more than 99.9% similar to EA181011 and 99.75% similar to ET080813. Both strains belong to the newly described species *Edwardsiella anguillarum* forming a separate branch close to *E. piscicida* together with EA011113 (Figure 1A). Multilocus Sequence typing (MLST) showed that the EA011113 strain is a new type, which is very close to ST12 and ST13 of the database^2^, both belonging to *E. anguillarum*. Figure 1B shows the phylogenetic tree constructed using the 62 strains of *Edwardsiella* spp. and suggests that EA011113 is similar to *E. anguillarum* strain EA181011 isolated from diseased grouper in Eilat, Israel and strain ET9 isolated in 2002 from *Pagellus bogaraveo* in Japan. *E. tarda*, *E. ictaluri*, and *E. hoshinae* have traditionally been the species that constitute the genus *Edwardsiella*. In the mid-60s, *E. tarda*, which is usually isolated from fresh or brackish water, was the first member of the genus (Ewing et al., 1965) able to cause disease in reptiles, fish and in rare cases humans (Hirai et al., 2015). The overwhelming majority of available *Edwardsiella* strains have been so far classified to *E. tarda* species. *Edwardsiella* strains have been previously isolated from both human stools and infected aquatic animals, however, due to their phenotypic variability and limited access to their genomic information, several of them have been falsely considered as *E. tarda*. During the last decade, research on *Edwardsiella* has been intensified and genome analyses gave rise to two more species, *E. piscicida* and *E. anguillarum*, that now include some of the strains previously thought to be *E. tarda* (Abayneh et al., 2012; Shao et al., 2015). The majority of the fish pathogenic strains belong to *E. piscicida* and only few, though highly virulent, to *E. anguillarum*. The analysis performed in the current study classifies the strain EA011113 isolated in Greece as a member of the *E. anguillarum* species. This is one of the first reports of this pathogen in the Mediterranean, since there is only one more isolate from this area that belongs to that species, that being strain 205/03 which was isolated in Spain from gilthead seabream, *Sparus aurata* (Buján et al., 2018a,b). The strain described here was isolated from diseased sharpsnout seabream, *Diplodus puntazzo* (Katharios et al., 2015), which belongs to the Sparidae family like gilthead seabream and *Pagellus bogaraveo* from which *E. anguillarum* has also been isolated in Japan. Interestingly, *Pagellus bogaraveo* is one of the fish species farmed in the same fish farm where the strain EA011113 was isolated and has been reported to be affected by Edwardsiellosis. Unfortunately, the identification of the strain was based on API (Analytical Profile Index) 20E therefore it was identified to genus level only (unpublished data). The MLST analysis performed in this study is in agreement with the very recent study on the evolution of the genus by Buján et al. (2018b). Strain EA011113 forms a monophyletic group with the strains 205/93, ET009 and DSM27202 (=ET080813) as in Buján et al. (2018b) but also with the strain EA181011 which was included in our analysis. This group is the new species, *E. anguillarum*.

### Prophage Detection and Characterization

According to the *in silico* analysis with PHASTER (Arndt et al., 2016), the genome of *E. anguillarum* EA011113 strain contains an intact prophage of 37 kb. In fact, spontaneous induction of the prophage took place while the bacteria were growing in liquid culture. The presence of a phage, designated as Edno5, was initially indicated by the formation of turbid plaques on EA011113 bacterial lawns following spot assay using serially diluted filtrates of the bacterial cultures.

Phage Edno5 was propagated using as host the lysogenized strain EA011113. This is peculiar since lysogenized bacteria have immunity to superinfection by the same phage. Similar cases are quite uncommon but have been reported in the literature. Bacteriophage Pf4, which has as a host *Pseudomonas aeruginosa* strain PAO1, was able to be converted into a superinfective form, producing plaques on its host’s bacterial lawn circumventing Pf4-mediated immunity (Rice et al., 2009). More recently it was reported that bacteriophages Str01 and Str03 were spontaneously released from *Streptococcus pyogenes* PCM 2855 and that very strain was subsequently used as their host for propagation (Harhala et al., 2018). In this case the authors examined whether the phages were prophages from the host bacteria but could not confirm their presence in the bacterial genome either by PCR or following whole genome sequencing of the host, suggesting that these were more likely environmental viruses that contaminated the original sample. Superinfection exclusion, which is defined as the prevention of a secondary infection from the same or a closely related phage of a pre-infected host, is necessary for the maintenance of viral latency, however, when ultravirulence (ability to grow in immune strains) occurs, viral latency can break down. Edno5 could be regarded as an ultravirulent phage that contains repressor protein of phage λ (CI). Using phage λ as a model, it has been shown that if superinfection inhibition and resistance against it coevolve in an arms race, then latency can be maintained (Berngruber et al., 2010). This could explain why although Edno5 is present as a prophage in the genome of the strain EA011113, it can still be efficiently proliferated using the same host.

**FIGURE 2:**
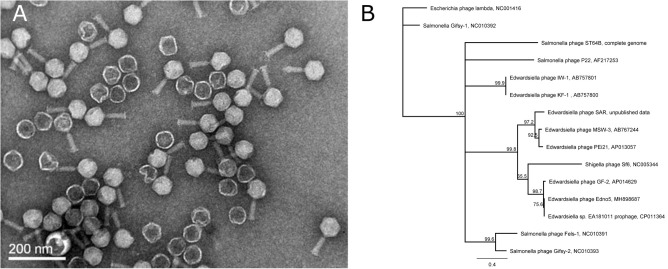
**(A)** Transmission (TEM) photograph of the phage Edno5 showing a Myoviridae morphology. **(B)** Phylogenetic tree performed according to Neighbor-Joining method (1,000 bootstraps) that includes bacteriophage Edno5 and 14 more Enterobacteria phages. Temperate phages GF-2, Edno5 and prophage of Edwardsiella sp. EA181011 are clustered together.

Evaluation of the induced bacteriophages’ morphology was performed by Transmission Electron Microscopy (TEM) observations classifying Edno5 to the Myoviridae family (Figure 2A). Measurements made on the obtained TEM pictures, revealed that the Edno5 bacteriophage has an icosahedral (isometric) capsid of 68.1 ± 3.5 nm equipped with a long rigid tail of 74.5 ± 4.1 × 18.2 ± 2.3 nm (*n* = 14). Endo5 virions resemble those of the *E. tarda* myoviruses GF-2 and MSW-3 since they were about 61 and 60 nm in capsid diameter and 81.6 and 80 nm in tail length, respectively (Kawato and Nakai, 2012; Yasuike et al., 2015).

The genome of phage Edno5 was found to contain 40,844 bp with a G+C content of 50.1% and 76 putative ORFs of which 42 encode hypothetical proteins (Supplementary Data S1). According to the bioinformatics analysis, the phage genome had no physical ends since the reads mapped to the beginning of the obtained contig could also be mapped to its end. Hence, the starting point of the phage contig obtained by the *de novo* assembly of the sequenced reads was arbitrarily placed. The circularization of the obtained contig revealed the formation of a ligase gene (gene 47) the amino-acid sequence of which matches that of a functional enzyme. Additionally, PhageTerm software indicated that Edno5 does not possess any termini and it is a permuted genome (Garneau et al., 2017). Given all previously mentioned evidence, bp 1 of the circularly permuted Edno5 phage genome was conventionally assigned as the first nucleotide of the intergenic region right before the putative small terminase subunit gene.

Edno5 is a temperate phage so it can undergo two distinct infection cycles: lytic or lysogenic. Lysogenized bacteria contain Edno5 as a prophage in their genome, because of the phage-encoded integrase (gene 34) activity, which facilitates the incorporation of Edno5 genome into the bacterial chromosome through site-specific recombination (Fogg et al., 2014). Although this process is fundamental for the temperate life cycle of the phage, maintenance of the prophage status and the consequent suppression of lysis is a multifaceted process, regulated by versatile repressor–antirepressor systems. Because of their significant importance to human health, Enterobacteria such as *Escherichia coli* and *Salmonella enterica* ser. Typhimurium and their corresponding phages lambda (Oppenheim et al., 2005; Casjens and Hendrix, 2015) and P22 (Prevelige, 2006), respectively, have been ideal model systems to study lysogeny. *Edwardsiella* is also a member of Enterobacteriaceae family, thus comparing these already studied systems to *E. anguillarum-*Edno5 phage system, reveals potentially conserved regulatory mechanisms. Specifically, gene homologs of *cI*, *cro*, and *cII* that are present in bacteriophage lambda are also found in the genome of Edno5 and relative *E. tarda* phage GF-2 (Yasuike et al., 2015). The lysogenic pathway can be interrupted by the expression of the *cro* gene (gene 53 in Edno5) which turns off early gene transcription during the lytic cycle. The genome of Edno5 contains an additional genomic area with regulatory role in the lysis/lysogeny decision, similar to that of bacteriophage P22 (Byl and Kropinski, 2000). This particular genomic region, designated as *ImmI*, contains 3 genes: *Ant*, *Mnt*, and *Arc*, all of them present in Edno5 indicating its potential contribution to the phage’s lysis/lysogeny decision. It should be stated that these findings were not tested under laboratory conditions but the common evolutionary pathway combined with both genetic and protein structure similarities with thoroughly studied Enterobacteria-phage model systems are strong indications for the function of *E. anguillarum*-Edno5 phage system. However, the role of the lysis/lysogeny regulatory gene sets in Edno5 needs to be further elucidated also by experimental work in the laboratory.

The presence of prophages in bacterial genomes is not considered only as a molecular time bomb but also an asset for coping under adverse environmental conditions (Paul, 2008). Lysogenic conversion is an important evolutionary process that may confer enhanced fitness to the prophage-carrying bacteria (Bondy-Denomy and Davidson, 2014). According to the *in silico* analysis on Edno5 genome that combined genetic and structural information of the ORFs, there are some genes that may be able to render beneficial features to *E. anguillarum*. N-6-adenine methyltransferase, which is encoded by gene 63, is an enzyme that interferes with the Restriction-Modification (RM) system of the host. Apart from protecting the viral DNA from cleavage by the host’s restriction enzymes, *N*-6-adenine methyltransferase-containing prophages have been reported to play an important role in lysogenic conversion of their hosts conferring them additional fitness and virulence properties (Low et al., 2001; Oakey et al., 2002; Kalatzis et al., 2017).

**FIGURE 3:**
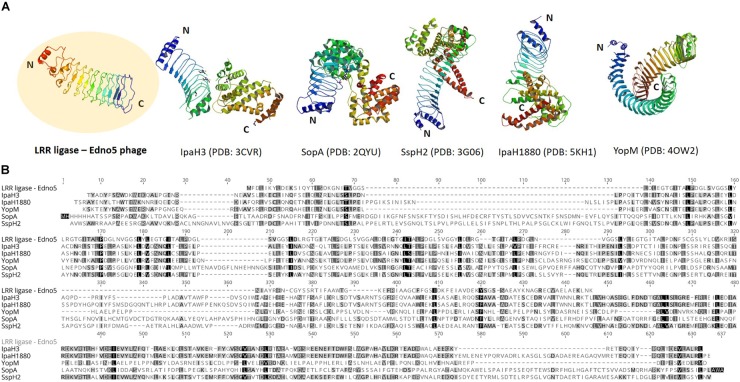
**(A)** Protein structure of LRR ligase encoded by Edno5 in comparison with 5 confirmed LRR-containing toxins from pathogenic Enterobacteria: IpaH3 (PDB: 3CVR) and IpaH1880 (PDB: 5KHI) from *Shigella flexneri*, SopA (PDB:2QYU) and SspH2 (PDB: 3G06) from *Salmonella enterica* ser. Typhimurium and YopM (PDB: 4OW2) from *Yersinia enterocolitica*, **(B)** Multiple alignment of the aa sequence of Edno5 LRR ligase with the 5 confirmed toxins, demonstrating in all of them the conserved LRR pattern.

According to bioinformatic analysis, gene 47 encodes a protein that contains a highly conserved Leucine-Rich Repeat (LRR) domain. The predicted protein structure of Edno5 gene 47 matches to a LRR ligase, resembling several confirmed virulent toxins of pathogenic Enterobacteria such as the *Shigella flexneri* effectors IpaH3 and IpaH1880 the *Salmonella enterica* ser. Typhimurium effectors SspH2 and SopA as well as the *Yersinia enterocolitica* effector YopM (Figure 3A,B). However, the size discrepancy between Edno5 LRR ligase (247 bp) and known enterobacteria effectors (∼600 bp) could justify the potential presence of an additional protein structure that could produce a functional final protein complex. Such effector proteins are characterized by E3 ubiquitin-protein ligase activity making them able to interfere with the host’s ubiquitination pathway. LRR-domains of these enzymes are responsible for recognizing the host substrates for ubiquitination as well as for interacting with a unique E3 ligase, hence assisting intracellular survival (Zhu et al., 2008; Quezada et al., 2009; Hicks and Galán, 2011; LaRock et al., 2015). The LRR-domain which was found in Edno5 is conserved in SopA, IpaH3, IpaH1880, SspH2, and YopM (Figure 3B) indicating that lysogenized *E. anguillarum* may interfere with its host’s ubiquitination pathways because of the Edno5 prophage, suggesting a plausible case of lysogenic conversion. *In silico* analysis showed that the genome of Edno5 is found also as a prophage in the genome of *Edwardsiella* sp. EA181011 (Reichley et al., 2015) with query coverage and identity, 99 and 97%, respectively. The second and last closest match was the *E. tarda* temperate phage GF-2 which at a query coverage of 75%, shares 92% identity with phage Edno5. Further phylogenetic analysis of bacteriophage Edno5 and 14 more Enterobacteria phages was conducted, using the terminase large subunit as an evolutionary marker since it is considered to be a conserved gene suitable for defining phage phylogenetic relationships. The obtained phylogenetic tree includes all 6 *Edwardsiella* phages that are present in the literature, the *Edwardsiella* prophage which has been found *in silico* in the genome of *Edwardsiella* sp. EA181011, bacteriophages P22 and lambda as well as 5 more *Salmonella* and *Shigella* phages. *Edwardsiella* phages SAR, MSW-3, PEi21, GF-2, Edno5, *Shigella* phage Sf6 and the prophage *Edwardsiella* sp. EA181011 were clustered together forming a monophyletic taxon, which is further divided into 3 subgroups. Bacteriophage Edno5 belongs to the temperate phages branch along with GF-2 and the prophage from *Edwardsiella* sp. EA181011 (Figure 2B). It can be mentioned that the life style of the bacteriophages constitutes a discriminating trait among the subgroups.

The virion analysis of Edno5 was complemented using the VIRFAM analysis, a recently proposed method that uses the “head-neck-tail” protein organization of phages (Lopes et al., 2014). It was shown that Edno5 belongs to type 7 cluster which is solely comprised of Myoviruses, therefore the molecular data corroborate to the observations made on the TEM pictures.

### Genomic Basis for the Bacterial Virulence of EA011113

**FIGURE 4:**
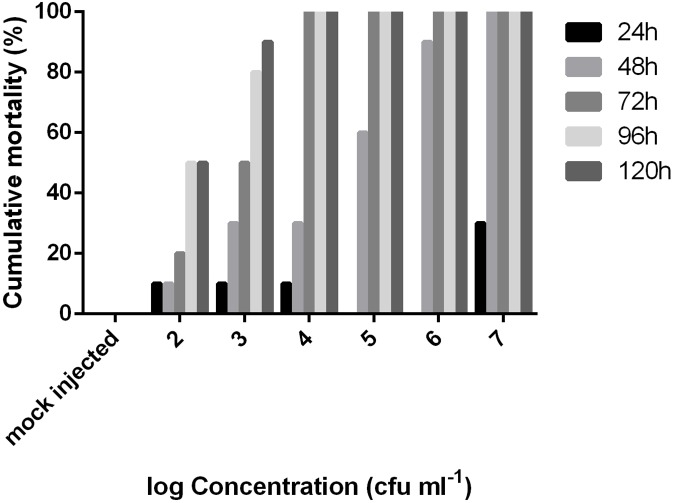
Cumulative mortality of adult zebrafish challenged with various concentrations of *Edwardsiella anguillarum* strain EA011113 administered via intraperitoneal injection over a 5-day period. LD50 value was calculated at 48 h post infection.

The LD_50_ value at 48 h post injection following probit analysis was 1.85 × 10^4^ cfu/fish (*R*^2^: 0.92, *F*: 36.38) or 6.12 × 10^4^ cfu/g. The mortality data of this experiment are presented for all concentrations in Figure 4. EA011113 is a highly pathogenic strain of *E. anguillarum* based on the results of the LD50 experiments in zebrafish. The LD50 values obtained are very low and indicate that even a few dozens of live cells can result in mortality within 2–3 days. Due to the fact that the majority of the studies on the *in vivo* virulence of *Edwardsiella* spp. were conducted before the description of the new taxons, almost all refer to *Edwardsiella tarda* although it is highly likely to be either *E. piscicida* or *E. anguillarum* making the comparisons difficult. Another difficulty is the inconsistency concerning the reporting of toxicity data in the virulence assays in different published studies. The strains AC35.1 and 9.8 (*E. piscicida*) had also very low LD50 values in turbot, *Scophthalmus maximus* 1.6 × 10^1^ and 1.2 × 10^2^ cfu/ml, respectively (Castro et al., 2008), while the typical and atypical (non-motile) strains of *E. tarda* had LD50 values ranging between 8.9 × 10^1^ and 2.6 × 10^7^ cfu/g body weight in 3 different species (*Seriola quinqueradiata*, *Paralichthys olivaceus*, and *Pagrus major*) (Matsuyama et al., 2005). It is highly likely that the atypical non-motile strain described in the latter study in Japan is *E. anguillarum* (Reichley et al., 2017). Moreover, *Edwardsiella* sp. isolated from diseased European seabass (*Dicentrarchus labrax*) in the Mediterranean coast of Spain had an LD_50_ of 1.4 × 10^8^ cfu/fish at 48 h post injection in 14-month old individuals of the same species. The type strain of *E. anguillarum* (ET080813) had an LD50 of 5.7 × 10^2^ cfu/g for turbot (Shao et al., 2015).

EA011113 has 3 clusters of T6SS and 2 clusters of T3SS as described in the type strain of *E. anguillarum* ET080813 (Shao et al., 2015). Pairwise and Mauve Alignment of the T3SSs and T6SSs of EA011113, EA181011, and ET080813 showed that these secretion systems have a high degree of sequence similarity (>99.8% for T3SS1 and T3SS2 and 99.8, 90.7, and 97.6% for T6SS1, T6SS2, and T6SS3, respectively) and synteny indicating that they are highly conserved among *E. anguillarum* strains, which is in accordance with the observations of Shao et al. (2015).

In *E. tarda*, a major virulence factor is the effector protein EvpP which is secreted by T6SS (Zheng and Leung, 2007; Wang X. et al., 2009). It has been observed that avirulent strains of *E. tarda* lack the gene encoding for EvpP whereas it is always present in virulent strains (Wang X. et al., 2009). In EA011113, EvpP is in the cluster of the T6SS1 (CGL57_12450). Furthermore, in *E. tarda*, EvP is regulated by the two-component system EsrA-EsrB (also present in EA011113, CGL57_17710, and CGL57_12705). Among the most important virulence factors reviewed for *E. tarda* (Park et al., 2012) are the autotransporter adhesin AIDA (CGL57_15010), which is used for attachment and penetration into the host cells, two hemolysins EthA (CGL57_17585) and EthB (CGL57_17585), which are regulated by the two-component system EsrA-EsrB, α-hemolysin regulator protein Hha (CGL57_17110) and ferric uptake regulator, Fur (CGL57_10755). Furthermore, quorum sensing (QS) plays an important role in the invasion and survival of *Edwardsiella* spp. intracellularly especially within the macrophages. QS controlled by QseB (CGL57_16255) and QseC (CGL57_16250) regulates virulence but also surface structures modifications (flagella and fimbriae) that may help bacteria’s adaptation in the intracellular niche (Wang et al., 2011). Another two-component system is also actively involved in virulence regulation; the PhoP-PhoQ (CGL57_06575 and CGL57_06570), which is used for sensing changes in temperature and Mg^2+^ concentration (Chakraborty et al., 2010) but also for the activation of T3SS and T6SS through the response regulator EsrB (Lv et al., 2012).

Ninety-four genes were predicted to encode virulence factors according to PATRIC annotation platform (Supplementary Data S2). As stated earlier, PATRIC combines 3 databases for the identification of virulence genes; PATRIC-VF from which 47 genes were identified, VFDB 21 genes and VICTORS 74. Secreted, periplasmic and outer membrane proteins related to virulence have been studied in *Edwardsiella piscicida* strain PPD131/90 using the *Tn*phoA transposon mutagenesis approach (Srinivasa Rao et al., 2003) followed by *in vivo* challenging. All of the genes that were experimentally shown to be related to virulence in *E. piscicida* were also present in EA011113 except orf20 which encodes a hypothetical protein (Table 1).

**Table 1:** Virulence genes experimentally confirmed in *E. piscicida*, their protein id, their orthologs in EA011113 and the % nucleotide similarity following pairwise alignment with the Geneious algorithm.

PPD130/91	Product	Protein id	EA011113 locus	% nucleotide identity
pstC	Peripheral membrane protein C	AAN05782.1	CGL57_07940	96.9
pstB	ATP binding protein B	AAN05784.1	CGL57_07930	96.6
pstS	Phosphate binding protein	AAN05781.1	CGL57_07945	95.1
isor	Iron sulfate oxidoreductase	ANL82723.1	CGL57_13235	94.9
orfA	Hypothetical	AAL01251.1	CGL57_15620	93.6
orf20	Hypothetical		–	
ssrB	Secretory system regulator	AAO52826.1	CGL57_17705	96.3
citC	Citrate lyase ligase	AAO52821.1	CGL57_04595	96.7
gadB	Glutamate decarboxylase isozyme	AAL82718.1	CGL57_05825	96.2
ompS2	Outer membrane protein	AAL82724.1	CGL57_00995	90.2
katB	Catalase precursor	AAL82719.1	CGL57_09535	93.6
astA	Arylsulfate transferase	AAK12109.1	CGL57_16650	76.9
fimA	Fimbrial protein	AAO52822.1	CGL57_17015	96.6
mukF	Killing factor	AAL827251	CGL57_06155	96.7

### Comparative Genomics Analysis Revealed Likely Genetic Basis for Lack of Motility of EA011113

**FIGURE 5:**
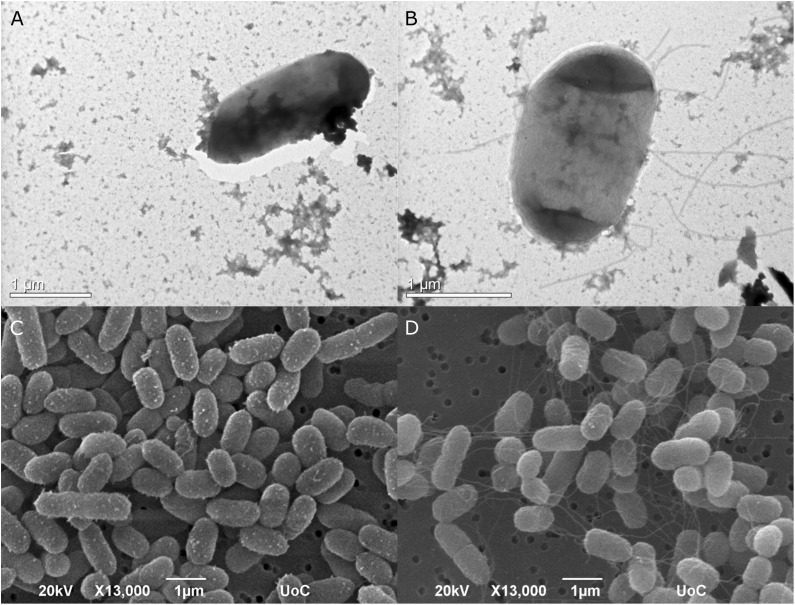
Transmission photomicrographs of negatively stained *E. anguillarum*. Strain EA011113 on the left where flagella are absent **(A)**, and the type strain of *E. anguillarum*, ET080813 bearing numerus peritrichous flagella **(B)**. Below: SEM photomicrographs of EA011113 **(C)** and ET080813 **(D)**.

The type strain of *Edwardsiella anguillarum* (ET080813) is motile, which is typical for most *E. tarda* strains isolated from fish, whereas non-motile strains are characterized as atypical (Matsuyama et al., 2005). The strain EA011113 described here together with the closely related EA181011 are non-motile. The motility of *E. anguillarum* is controlled by peritrichous flagella (Shao et al., 2015). Bacterial flagella are multicomponent organelles that their biosynthesis and function require the function of more than 50 genes. Flagellar-related genes in EA011113 are found in three gene clusters. Following Mauve alignment of the three cluster from the two non-motile strains, EA011113 and EA181011 and the motile ET080813 a deletion of 36 nt was found in the gene encoding the flagellar biosynthetic protein FlhB. It has been shown that a functional *flhB* gene is required for the formation of the rod structure of the basal body of the flagellar apparatus (Minamino et al., 1994; Williams et al., 1996) and the observed deletion may have resulted in the non-motile phenotype of strains EA011113 and EA181011. This finding is in accordance with the lack of flagella in the EA0111113 strain as opposed to the peritrichous flagella of the type strain ET080813, which were described previously but also observed by TEM and SEM in the current study (Figure 5). However, the association of the 36 nt deletion in the *flhB* of the non-motile strains with the loss of flagella needs to be verified experimentally.

## Conclusion

Current study reclassifies the bacterial strain EA011113 that was isolated in Greece infecting cultured sharpsnout seabream, *D. puntazzo* as *E. anguillarum*. The genomes of strain EA181011 that was isolated from diseased grouper in Israel and strain ET9 that was isolated from *P. bogaraveo* in Japan, showed very high levels of similarity with *E. anguillarum* strain EA011113, indicating its quite broad geographical distribution. However, unlike the Chinese *E. anguillarum* type strain (ET080813), the Greek and the Israeli strains are non-motile possibly due to a 36 nt long deletion in the flagellar biosynthetic protein gene (flhB). *In vivo* infection trials in zebrafish along with *in silico* analysis of the bacterial genomes demonstrated that *E. anguillarum* strain EA011113 is a highly virulent strain that needs to be considered among causative agents of edwardsiellosis. The studied strain contains an intact, inducible prophage that according to genomic analysis it may confer significant fitness to its host through lysogenic conversion. Following the bacterial host, this phage has a wide geographical distribution too and may play a significant evolutionary role the species since it can be found both as prophage and as a free virion.

## Author Contributions

PK designed the study and carried out the genomic analysis of the bacterium. QW contributed in the genomic analysis. CK isolated the phage and carried out the laboratory work for the study. MP and PK carried out the *in vivo* studies. PGK contributed with the *in silico* genomic analysis of the prophage. PK and PGK wrote the manuscript which was reviewed by all authors.

## Conflict of Interest Statement

The authors declare that the research was conducted in the absence of any commercial or financial relationships that could be construed as a potential conflict of interest.

## Footnotes

1 https://pubmlst.org/edwardsiella/

2 https://pubmlst.org/edwardsiella/

## Supplementary Material

DATA S1 Gene content of bacteriophage Edno5 and its genomic map.

DATA S2 List of virulence factors of the strain EA011113.
